# Assessment of cardioprotective activity of a nitrate enriched food-based polyherbal formulation using experimental myocardial infarction in rats

**DOI:** 10.3389/fcvm.2026.1857264

**Published:** 2026-07-10

**Authors:** Sruthi Ramagiri, Hemraj Singh, Rajeev Taliyan, Yogendra Kumar Choudhary, Iwin Joseph Benny

**Affiliations:** 1 Department of Pharmacy, Birla Institute of Technology and Science (BITS Pilani), Rajasthan, India; 2 JSS College of Pharmacy, Ooty, Tamil Nadu, India; 3 Arjuna Natural Pvt. Ltd., Keezhmad, Aluva, Kerala, India

**Keywords:** *Amaranthus*, BCM-95, curcuminoids, dietary nitrate, myocardial ischemia, oxystorm, troponin-I

## Abstract

**Introduction:**

Recently, Myocardial infarction (MI) became the foremost causes of mortality globally. Growing modern pharmacological evidences indicate that food based herbal formulations can offer promising therapeutic benefits in the management of cardiovascular diseases.

**Materials and methods:**

In the current study, we investigated the therapeutic potential of a newly developed nitrate-enriched extract from *Amaranthus* leaves, Oxystorm, and its blend with bioactive food constituent, curcuminoids enriched turmeric extract (BCM-95) in an *in vivo* model of MI, induced by the ligation through left anterior descending coronary artery (LAD). The model caused significant alterations in cardiac function and structure. In both models, Wistar rats were randomly divided into different groups (three different control groups namely vehicle, sham, and LAD), a positive control group (verapamil), low-dose Oxystorm, and high-dose Oxystorm treatment groups, and a synergistic group administered with Oxystorm and turmeric extract. Following reperfusion, samples were collected to determine cardiac injury biomarkers; LDH, CK-MB, myoglobin, and troponin I. Moreover, inflammatory biomarkers (interleukin-6 and TNF-α) and endogenous oxidative stress markers were assessed.

**Results:**

LAD-induced MI significantly increased LDH, CK-MB, oxidative stress, and cardiac inflammation. However, treatment with Oxystorm and its turmeric blend alleviated levels of lactate enzyme, CK-MB, troponin-I, interleukins, and malondialdehyde, which reduced infarct size, though enhanced antioxidant defense (GSH and SOD).

**Conclusion:**

The enriched dietary nitrate content in Oxystorm, when combined with polyphenol-rich turmeric extract (BCM-95), showed cardioprotective potential, which may be associated with nitrate/NO-related mechanisms, attenuation of oxidative stress, and suppression of inflammatory pathways. However, nitric oxide bioavailability and related signaling pathways were not directly assessed in the present study. Taken together, the pioneer observations, suggest that Oxystorm and its turmeric combination, may serve as adjuvants for the management of ischemia and related cardiac diseases.

## Introduction

Cardiovascular disease (CVD) has been considered as a predominant reason of life threatening worldwide (1). Recently reported that 19.8 million people suffered from cardiac dysfunction in 2022, which will be projected to rise to an alarming 35.6 million by 2050 from 20.5 million in 2025 (2). Varying lifestyles, dietary habits, and physical inactivity become significant contributors to heart disease (3). During the scenario, reduced blood supply to the heart decreases the delivery of oxygen and nutrients, which impairs the cellular functions. However, timely reperfusion is essential to prevent further damage and restore normal cardiac physiology (4).

The enhanced risk of cardiovascular disease with aging is primarily attributed to antagonistic changes in arteries, including vascular endothelial dysfunction and increased stiffness of large elastic arteries (5). Reactive species (ROS or RNS) such as hydroxyl radicals, singlet oxygen, hydrogen peroxide, peroxynitrite, superoxide radicals, and nitric oxide, significantly contribute to myocardial injury (6). A crucial mechanism involved in the age-related endothelial dysfunction is the reduced bioavailability of the vasoprotective and vasodilatory molecule, nitric oxide (NO). As a diverse signaling molecule, nitric oxide plays an essential role in regulating cardiovascular homeostasis. Impairment of NO signaling pathway is constantly allied with augmented disease conditions. The age-related deterioration in NO bioavailability is partly determined by oxidative stress, which has been characterized by an imbalance between reactive oxygen species and antioxidant defense mechanisms (7, 8). Besides drug therapy, lifestyle changes, dietary modifications, and supplementation with an antioxidant rich herbal formulation have demonstrated protective effects against CVDs without compromising their therapeutic value and by considering the importance of dietary intake rather than endogenous production. Further evidences suggest that bioactive substances may serve as potential therapeutic candidates against various chronic diseases, and their alternative formulations may boost their bioavailability (9).

Vegetables are considered important components of diet as they contain multi array of bioactive compounds (10). Beetroot, in particular, has been reported earlier for its high nitrate content and subsequent cardioprotective effect on hypertensive individuals. The study demonstrated the role of nitrate in ameliorating endothelial function, reducing arterial stiffness and promoting smooth muscle relaxation (11). Beet root juice also has been investigated for their natural and safe potential in reducing myocardial infarction caused by ischemic reperfusion injury (12).

*Amaranthus* genus, coming under Amaranthaceae family, encompasses a group of widely consumed leafy vegetables, have emerged as an alternative dietary nitrate source with potential applications in cardiovascular health and workout performance (13, 14). In addition to enriched content of nitrate, previous reports have emphasized the presence of flavonoids, phenolics, minerals and bioactive pigment in *Amaranthus*, all of which may contribute to nitric oxide production and subsequent antioxidant activity (15). A previous study has emphasized the enhancement of nitrate (NO_3_ˉ) and nitrite (NO_2_ˉ) levels in supporting the performance of people involved in strong physical activities or sports (16). Moreover, reduced sugar content also maintains substantial nitrate concentration. Previous clinical pharmacokinetic studies established that oral intake with *Amaranthus* extract markedly elevated nitrate/nitrite concentrations in both saliva and plasma (17). The observations further demonstrated increased nitrate/nitrite availability following supplementation, which is consistent with the established nitrate–nitrite–nitric oxide pathway (18). Furthermore, a recent cross over placebo-controlled study indicated species dependent variations in nitrate/nitrite bioavailability among *A. hypochondriacus*, *A. tricolor*, and *A. hydridus* (19).

Furthermore, *Amaranthus* species exhibits a plethora of biological activities, including the mitigation of oxidative stress and inflammation, modulation of gut microbiota, management of vascular function and lipid metabolism, as well as the amelioration of hyperglycemia, and hypertension (20, 21). The distinct structural traits of phytoconstituents, such as amaranthine, *β*-xanthin, betalain, *β*-cyanin, as well as carotenoids, and vitamin C, may also enhance these functional activities (22, 23). In mammalian tissues, nitrites and nitrates are reduced to nitric oxide (NO), which elevates its availability within the vasculature, subsequently improving endothelial function and maintaining overall cardiovascular health. Recently, the growing interest in dietary nitrite and nitrates may be due to improved understanding of their key role in vascular homeostasis, through regulation of blood flow and protection against cardiovascular dysfunction (24). Consequently, nitrate and nitrite-rich foods such as *Amaranthus* have attracted attention as a potential functional dietary element for promoting cardiovascular well-being. Oxystorm® is a nitrate enriched extract from *Amaranthus* leaves, which derived its applications in reducing fatigue and help to maintain an optimal physiological function during hypoxia conditions through the supply of nitric oxide (Oxystorm—The nitrate supplement you need—Curegarden Nutraceutical).

Curcuminoids (curcumin, demethoxy curcumin, and bisdemethoxy curcumin), dietary polyphenolic compounds from *Curcuma longa*, is recognized to exhibit numerous beneficial biological activities, including antioxidant, hepatoprotective, anti-proliferatory, anti-inflammatory, hypouricaemic, anti-pruritic, anti-apoptosis, analgesic, anti-dyspeptic, anti-anxiety, anti-depressant, and anti-arthritic effects. Similarly, a curcuminoids-based formulation enriched with antioxidant phenolic compounds has been shown to reduce myocardial fibrosis by controlling the expression of angiotensin II receptors (AT1 and AT2) in angiotensin II-treated rats and ASD-Cur, toward cardiac failure (25). BCM-95® is a clinically validated, extensively studied, and a highly bioavailable form of turmeric extract, which combines curcuminoids with turmeric essential oil enriched with ar-turmerone. This specific composition markedly promotes the absorption, and the efficacy of curcumin compared to traditional curcumin extracts, thereby surpassing the main limitation of restricted bioavailability allied with native curcumin formulations. This combination has been previously reported to contain 95% of total curcuminoids and turmeric essential oil, with curcuminoids and curcumin constituting not less than 88% and 68%, respectively (26). This synergistic combination particularly with ar-turmerone, contributes to enhanced pharmacokinetic and therapeutic activities. Numerous clinical and preclinical studies have also established its antioxidant, anti-inflammatory, neuroprotective, and immunomodulatory activities. Moreover, BCM-95 has shown promising value in circumstances associated with oxidative stress, metabolic disorders, arthritis, multiple sclerosis, and cognitive dysfunctions (27–29). The superior bioavailability and wide spectrum of biological activities, BCM-95 is promoted as a principal turmeric formulation for both therapeutic and nutraceutical applications.

Considering the multifaceted effects of *Amaranthus* and curcuminoids enriched turmeric extract, especially as an effective nitrate-rich phytoconstituent and a known antioxidant and an anti-inflammatory polyphenol enriched botanical ingredient, their combination may represent a promising strategy for the management of myocardial ischemia-reperfusion injury. While both Oxystorm and BCM-95 have independently established for their noteworthy roles in various experimental designs, scientific indications in managing myocardial ischemic reperfusion injury remains scarce and warrants further exploration. To our best knowledge, no earlier study has been scientifically investigated the cardioprotective effects of a nitrate-enriched *Amaranthus*-based formulation in combination with curcuminoid-enriched turmeric extract in contrary to myocardial ischemia-reperfusion. Therefore, the current study was intended to assess, for the first time, the therapeutic potential of Oxystorm and BCM-95 combination in experimental cardiac dysfunction in rats, along with explicating its probable underlying mechanisms.

## Materials and methods

### Chemicals and reagents used

2,3,5-Triphenyltetrazolium chloride (TTC) (Cat # 17779), sodium dodecyl sulphate (Cat # 71725), acetic acid (Cat # A 6283), thiobarbituric acid (Cat # T 5500), tetramethoxypropane (Cat # 108383), glycine buffer solution (Cat # G 5418), epinephrine (Cat # E 4250), 5, 5′-dithiobis-(2-nitrobenzoic acid) (Ellman's reagent) (Cat # D 8130), isoflurane (Cat # 792632), formic acid (Cat # 5.43804), tetrabutyl ammonium hydroxide solution (Cat # 178780), Folin Ciocalteu reagent (Cat # F9252), and sodium nitrite (Cat # 237213) were procured from Sigma-Aldrich (St. Louis, MO, USA). HPLC grade methanol (Cat # 1.06018), acetonitrile (Cat # 1.00030), potassium nitrate (Cat # 1.05063), USP grade reference standards, curcumin (Cat # 1151855), demethoxy curcumin (Cat # 1173100) and bisdemethoxy curcumin (Cat # 1075305) were purchased from Merck India Ltd (Mumbai, Maharashtra, India). ELISA LDH kit (Cat # 1001260) and protein kit (Cat # 1001291) were obtained from Spinreact (C/Arago 208, 5-2, 08011, Barcelona, Spain), and ELISA CK-MB kit (Cat # CKB1222) was purchased from Coral Clinical System (Verna, Goa, India). Troponin I kit (Cat # VMD49) was obtained from Vitrosens Biotechnology Inc., Serifali, Istanbul. Millipore water was obtained from Milli-Q system (Millipore, Billerica, MA, USA). All other chemicals used were of standard analytical grade.

### Plant material and extraction

Oxystorm, an *Amaranthus* extract enriched with dietary nitrate, and curcuminoids enriched turmeric extract (BCM-95/Curcugreen) in different doses were used in the present study. Oxystorm was prepared by extracting fresh leaves of *Amaranthus sp.* with millipore water at 55 °C. The aqueous extract was filtered, pasteurized for 2 h at 90–100 °C, and then spray-dried (inward temperature 180 ± 5 °C; outward temperature 90 ± 5 °C). However, BCM-95 is a dual proprietary blend of curcuminoids (curcumin, demethoxy curcumin, and bisdemethoxy curcumin) enriched with turmeric essential oil containing ar-turmerone (Antony, 2008). Both were prepared by Arjuna Natural Pvt Ltd., Kerala, India. The resultant dried powders were uniformly blended and sieved to ensure homogeneity separately. The final products were subsequently used for the study.

### HPLC methods for the analysis of nitrates in Oxystorm and curcuminoids in enriched turmeric extract (BCM-95)

#### Preparation of standard solutions

Accurately weighed potassium nitrate (81.53 mg) was added with 50 mL millipore water into a 100 mL volumetric flask and sonicated for 5 min. Diluted the solution to volume with water and mixed well to obtain the standard stock solution. 1 mL of the standard stock solution was then pipetted into a 50 mL volumetric flask and diluted to volume with water. The resultant working standard solution was filtered through a 0.22 µm syringe filter prior to HPLC analysis.

For curcuminoids analysis, around 10 mg of individual curcuminoid reference standards were accurately weighed and transferred into a 10 mL volumetric flask. The standard mixture was dissolved in methanol and diluted to volume with the same solvent to obtain the standard stock solution. An aliquot of the stock solution was further diluted with methanol to obtain a final concentration of 50 µg/mL. The solution was filtered through a 0.2 µm PTFE membrane syringe filter and used for HPLC analysis.

#### Preparation of sample solutions

For nitrate analysis, accurately weighed Oxystorm (250 mg) was dissolved in 50 mL millipore water and sonicated the mixture in an ultrasonic water bath for 20 min. Pipetted out 2 mL of this solution into a 25 mL standard flask, made up to the volume with water and mixed well. For curcuminoid analysis, approximately 25 mg of turmeric extract (BCM-95) was accurately weighed and transferred into a 25 mL volumetric flask. The sample was dissolved and diluted to volume with HPLC-grade methanol to obtain the standard stock solution. The stock solution was further diluted with methanol to obtain a final concentration of 50 µg/mL. The prepared solutions were then filtered separately through 0.2 µm PTFE membrane syringe filters before chromatographic analyses.

#### Chromatographic conditions

The nitrate content in Oxystorm was analysed using a Waters ultra-performance liquid chromatography (UPLC) system (Waters Corporation, Milford, MA, USA) equipped with a photodiode array (PDA) detector. Chromatographic separation was achieved using a Waters ACQUITY UPLC BEH C-18 column (2.1 × 100 mm, 1.7 µm particle size). Gradient elution was carried out using mobile phase A [tetrabutyl ammonium hydroxide (TBAH) buffer, pH 2.5], B (acetonitrile), and C (methanol). Mobile phase composition was initiated with 92% A and 8% B. The gradient run was further adjusted to 48% A, 8% B, and 44% C, followed by equilibration to initial concentration. For the analytical run of 18 min, the flow rate was maintained at 0.1 mL/min and increased to 0.2 mL/min during column re-equilibration.

For curcuminoid analysis, the analysis was performed using a Waters high-performance liquid chromatography system equipped with a PDA detector. Separation was achieved on a Waters SunFire C-18 column (150 × 4.6 mm, 5 µm particle size). Chromatographic separation was carried out under isocratic conditions using a mobile phase consisted of acetonitrile and 0.1% formic acid in water (45:55, v/v). The flow rate was maintained at 1 mL/min, and detection was performed at 420 nm. The injection volume was 20 µL, and the total run time was 20 min.

### HPLC methods for the quantification of nitrates in Oxystorm and curcuminoids in enriched turmeric extract (BCM-95)

Quantification of nitrates in Oxystorm and curcuminoids in the enriched turmeric extract was performed by utilising HPLC methods similar to those specified above for standardisation. Nitrate content in Oxystorm and curcuminoids in turmeric extract (BCM-95) were calculated by comparing the peak areas of respective analytes with corresponding reference standards using the following equations [Equations (1)–(5)]. Nitrate content in Oxystorm was calculated using Equation (1), whereas total curcuminoid content in enriched turmeric extract was calculated using Equations (2)–(5).

$$\begin{array}{rl} & \mathrm{Percentage}\;\mathrm{of}\;\mathrm{nitrate}\\ & =\frac{\mathrm{area}\;\mathrm{of}\;\mathrm{nitrate}\;\mathrm{peak}\;\mathrm{in}\;\mathrm{sample}\;x\;\mathrm{weight}\;\mathrm{of}\;\mathrm{standard}\;x\;\mathrm{purity}\;\mathrm{of}\;\mathrm{standard}}{\mathrm{area}\;\mathrm{of}\;\mathrm{nitrate}\;\mathrm{peak}\;\mathrm{in}\;\mathrm{standard}\;x\;\mathrm{weight}\;\mathrm{of}\;\mathrm{the}\;\mathrm{sample}} \end{array}$$ (1)

$$\mathrm{Percentage}\;\mathrm{of}\;\mathrm{curcumin}=\frac{\mathrm{area}\;\mathrm{of}\;\mathrm{curcumin}\;\mathrm{peak}\;\mathrm{in}\;\mathrm{sample}\;x\;\mathrm{weight}\;\mathrm{of}\;\mathrm{standard}\;x\;\mathrm{purity}\;\mathrm{of}\;\mathrm{standard}}{\mathrm{area}\;\mathrm{of}\;\mathrm{curcumin}\;\mathrm{peak}\;\mathrm{in}\;\mathrm{standard}\;x\;\mathrm{weight}\;\mathrm{of}\;\mathrm{the}\;\mathrm{sample}}$$ (2)

$$\mathrm{Percentage}\;\mathrm{of}\;\mathrm{demethoxycurcumin}=\frac{\begin{array}{c} \mathrm{area}\;\mathrm{of}\;\mathrm{demethoxycurcumin}\;\mathrm{peak}\;\mathrm{in}\;\mathrm{sample}\;x\;\;\\ \mathrm{weight}\;\mathrm{of}\;\mathrm{standard}\;x\;\mathrm{purity}\;\mathrm{of}\;\mathrm{standard}\; \end{array}}{\begin{array}{c} \mathrm{area}\;\mathrm{of}\;\mathrm{demethoxycurcumin}\;\mathrm{peak}\;\mathrm{in}\;\mathrm{standard}\;x\;\;\\ \mathrm{weight}\;\mathrm{of}\;\mathrm{the}\;\mathrm{sample}\; \end{array}}$$ (3)

$$\mathrm{Percentage}\;\mathrm{of}\;\mathrm{bisdemethoxycurcumin}=\;\frac{\begin{array}{c} \mathrm{area}\;\mathrm{of}\;\mathrm{bisdemethoxycurcumin}\;\mathrm{peak}\;\mathrm{in}\;\mathrm{sample}\;x\;\;\\ \mathrm{weight}\;\mathrm{of}\;\mathrm{standard}\;x\;\mathrm{purity}\;\mathrm{of}\;\mathrm{standard}\; \end{array}}{\begin{array}{c} \mathrm{area}\;\mathrm{of}\;\mathrm{bisdemethoxycurcumin}\;\mathrm{peak}\;\mathrm{in}\;\mathrm{standard}\;x\;\;\\ \mathrm{weight}\;\mathrm{of}\;\mathrm{the}\;\mathrm{sample}\; \end{array}}$$ (4)

$$\begin{array}{rl} & \mathrm{Total}\;\mathrm{content}\;\mathrm{of}\;\mathrm{curcuminoids}\;\mathrm{in}\;\mathrm{the}\;\mathrm{given}\;\mathrm{sample}\\ & =\mathrm{Sum}\;\mathrm{of}\;\mathrm{total}\;\mathrm{percentage}\;\mathrm{content}\;\mathrm{of}\;\mathrm{curcuminoids}\;\\ & \left(\mathrm{curcumin}+\mathrm{demethoxy}\;\mathrm{curcumin}+\mathrm{bisdemethoxy}\;\mathrm{curcumin}\right) \end{array}$$ (5)

### Evaluation of total phenolic and flavonoid contents in enriched turmeric extract (BCM-95)

The amount of total phenolics in the turmeric extract samples were determined using the Folin-Ciocalteu reagent method (30). 1 mL of Folin-Ciocalteu reagent and 12 mL of sodium carbonate (7% w/v) were added to 2 mL of the sample solution (1 mg/mL). The resultant mixture was incubated at ambient condition for 20 min. The absorbance of the sample was measured at 760 nm using a UV-visible spectrophotometer (Lambda 365+, Perkin Elmer, Waltham, MA, USA). A standard calibration graph of gallic acid (GA) was plotted with absorbance of different concentrations of gallic acid (3–10 mg/mL) along *X*-axis and concentration along *Y*-axis. The results were expressed as g gallic acid equivalents (GAE)/100 g extract.

The formation of flavonoids—aluminium complex with an absorption maximum at 510 nm was used for the evaluation of total flavonoid content in turmeric extract (31). For the evaluation, 1 mL of the sample in methanol (10 mg/mL) was added to 4 mL of distilled water followed by the addition of 3 mL 5% sodium nitrite solution. After incubating the solution at 25 °C for 5 min, 0.3 mL of 10% aluminium chloride solution was added and kept for 5 min. The reaction mixture was treated with 2 mL of 1 M sodium hydroxide solution. Finally, the reaction mixture was diluted to 1 mL with water and absorbance was measured at 510 nm using a Lambda 365+ (Perkin Elmer, Waltham, MA, USA) spectrophotometer. A standard calibration graph of catechin was prepared by plotting the absorbance of different concentrations of catechin (20–100 mg/mL) prepared in methanol along the *X*-axis and concentration along the *Y*-axis. The estimations were carried out in triplicates. The results were expressed as g catechin equivalent (CE)/100 g extract.

### Experimental animals and protocol

Adult Wistar rats (weighing 150–200 g) have been procured from Birla Institute of Technology and Science (BITS), Pilani, Rajasthan, India. The experimental procedures complied with the guidelines of the Committee for the Control and Supervision of Experiments on Animals (CCSEA), Government of India (https://ccsea.gov.in/Content/54_1_ACTS,RULESANDGUIDELINES.aspx). The experiments were conducted following approval by the Institutional Animal Ethics Committee (IAEC/RES/22/08). The animals were sustained at optimum laboratory conditions with a temperature of 22 ± 2 °C and room humidity of 60 ± 10% along with a 12:12 h light/dark cycle. They were nourished with a standard diet and had free access to filtered water. In both models, rats were randomly assigned to 7 groups, each consisting of 8 animals, with equal distribution of male and female rats to minimize sex allocation bias. Both male and female rats were included in equal numbers across all experimental groups. Preliminary sex-wise comparisons of the measured endpoints were performed within each experimental group prior to pooled analysis. No statistically significant differences between male and female animals were observed for the assessed biochemical, inflammatory, oxidative stress, or infarct size parameters. As no consistent sex-specific pattern was detected, the data were pooled for subsequent statistical analyses. The study groups included a vehicle control group receiving 0.5% carboxymethyl cellulose (CMC), a sham control group, a LAD control group subjected to ischemia-reperfusion injury alone, and a verapamil-treated LAD group (10 mg/kg) served as the positive control.

For both preventive and treatment models, Oxystorm was administered at two dose levels: a low dose (45 mg/kg in the preventive model and 90 mg/kg in the treatment model) and a high dose (90 mg/kg in preventive model and 180 mg/kg in treatment model), following ischemia–reperfusion injury. In addition, a combination treatment of Oxystorm and curcuminoids enriched turmeric extract (BCM-95) was evaluated for synergistic effects. In this combination group, Oxystorm and curcumin were administered at 90 mg/kg each in the preventive model, whereas in treatment model both were administered in the ratio 2:1 (180 mg/kg Oxystorm and 90 mg/kg BCM-95 respectively).

During dosing period, the animals were clinically examined once daily for death incidence and disease burden. Common clinical signs (like excessive grooming, less movement, crouched position, twitching, lack of balance in walking, and low rear or excessive grooming) were also recorded during the post-treatment period.

### Dosing schedule

For the preventive model, Oxystorm (low dose and high dose) was prepared in water and administered orally. Curcuminoids enriched turmeric extract (BCM-95) (90 mg/kg), prepared in dimethyl sulfoxide due to its limited aqueous solubility, was co-administered with Oxystorm (90 mg/kg), which was dissolved in water, as a synergistic treatment via the oral route for a duration of thirty days. On 31st day, reperfusion was performed for 60 min, post 45 min of ischemia induction by ligation through the left anterior descending coronary artery.

**Figure 1:**
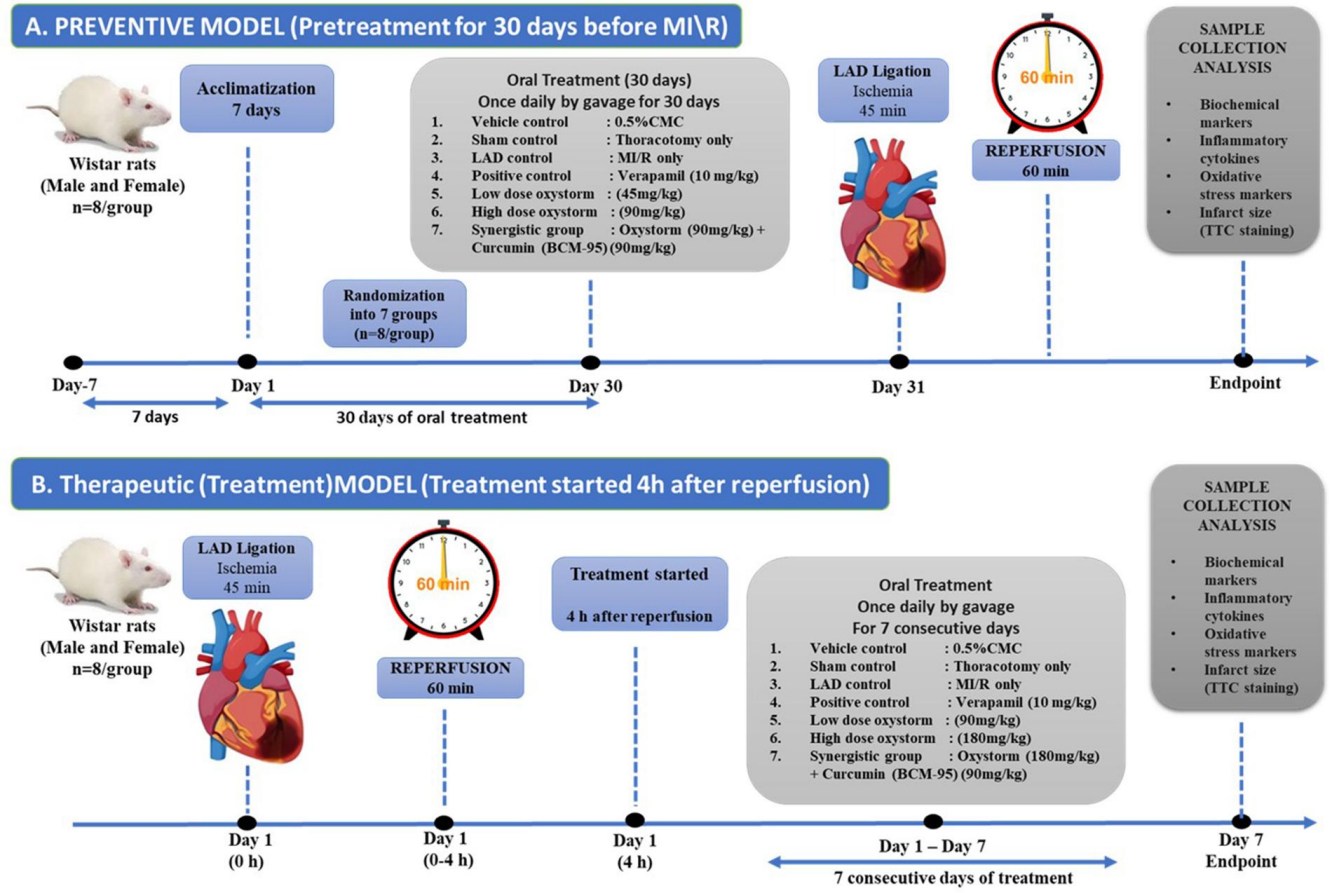
Experimental timeline and dosing schedule for the preventive and therapeutic models of myocardial ischemia/reperfusion (MI/R) injury in Wistar rats. **(A)** In the preventive model, animals received daily oral administration of test formulations for 30 consecutive days before induction of MI/R injury by LAD coronary artery ligation (45 min ischemia followed by 60 min reperfusion). **(B)** In the therapeutic model, treatment was initiated 4 h after reperfusion and continued once daily for 7 consecutive days. Animals were subsequently sacrificed for infarct size determination, biochemical marker analysis, assessment of inflammatory cytokines, and assessment of oxidative stress. Figure was created by the authors using Microsoft PowerPoint. Graphical elements were adapted from publicly available online resources and assembled for illustrative purposes.

In the treatment model, ischemia was induced for 45 min followed by reperfusion for 7 days. Reperfusion was established by the reoccurrence of a pink-red color on the anterior wall of the left ventricle within 20 s after removal of ligature. The organ was reinstated to its original position and sutured. Tested drug administration was initiated four hours after reperfusion, and continued once daily until day 7. During the experimental duration, the cardiac function parameters were recorded at predetermined intervals. Afterward, the animals were sacrificed by using cervical dislocation method through cervical vertebrae using a steel rod, and their hearts were excised for different studies. A schematic representation of the preventive and therapeutic experimental workflow was presented in Figure 1.

### Experimental procedure

Ischemic reperfusion injury was achieved by left anterior descending artery (LAD) ligation model. Briefly, each rat was anesthetized individually via an induction chamber using 5% isoflurane. The animals were then intubated and mechanically ventilated utilising an animal respirator expelling 2% isoflurane in oxygen (0.4 L/min). Afterward, a thoracotomy was performed by a tilted incision along the left sternal edge positioned around 1 cm length and 2 mm laterally below the junction of body and left front leg. The incision was achieved carefully to avoid damage to the superficial thoracic vein, ensured that the lateral end approached the vessel without any penetration.

An incision on thoracic muscle was made to represent the underlying ribs, which allows the visualization of the ribs and the inflated lung through the semitransparent wall. The cavity was then opened by making an incision of 6–8 mm at the fifth intercostal position and was located approximately 2 mm from the sternal border. Sterilized chest retractors were inserted into the incision and mildly expanded to a width of around 8–10 mm, to avoid the contact. At the stage, the retractors were pinned to the surgical platform, in which the heart became visible. The pericardium was gently grasped with curved forceps for making incision, and folded back behind the retractors. During the procedure, the maneuver lifted the lung far from the heart. Following this, the left anterior descending coronary artery was then observed on the surface of the heart using a dissection microscope for robust localization.

To enable the ligation, a sterile cotton ball fragment (approximately 1–2 mm in diameter) was placed between the left atrium and ventricle, which helped to elevate the left atrium and subsequently improve visualization. The LAD was ligated 4–5 mm below the tip of the left auricle using a pulmonary trunk as an anatomical marker. A traumatic needle was used to permit a 5–0 silk suture underneath the LAD, and the ends were passed through a small vinyl tube to make a snare. The needle was inserted with more precision to avoid damage to the artery, if any. In the outset of accidental damage to LAD, the needle was detached and was repaired to arrest bleeding, if hemostasis could not be achieved, the animal was humanely euthanized. A movable double knot was formed, made a 2–3 mm opening through an insertion of PE tubing (2–3 mm). The loop was further tightened around both the arteries and secured with an additional slipknot, to avoid excessive pressure on the ventricular wall. Successful occlusion was established by the quick appearance of a pale color in the anterior wall of left ventricle after a few seconds of ligation.

Gently removed the retractor and momentarily closed the incision using a bulldog clamp. The rat continued on the ventilator throughout the duration of LAD occlusion. Ischemia was maintained for 45 min, followed by reperfusion for 7 days after removal of the ligature. At the later ischemic period, removed bulldog clip and inserted retractors to re-expose the suture. The knot was removed, and the tubing was removed to ensure reperfusion. The heart was allowed to return to its normal position and sutured thereafter. After restoration of the chest cavity, post-operative analgesics were provided using buprenorphine (0.05 mg/kg, subcutaneous injection) every 12 h for 24 h, in accordance with established postoperative pain management protocols (32).

### Measurement of myocardial infarct size

At the end of 7-day reperfusion study, rats were anesthetized, and the hearts were quickly excised, frozen at −20 °C, and cut horizontally into 4–5 sections. The sections were incubated using 2,3,5-triphenyltetrazolium chloride (TTC) [2% in phosphate buffer (pH 7.8)] at 37 °C for 30 min (33), followed by the colour fixation with paraformaldehyde and sections were captured with a digital camera. TTC stains enabled the distinguished identification of viable myocardium, which appeared red, whereas the infarcted region remained unstained (white). TTC stained and unstained areas were demarcated, and analyzed using an Image J (NIH, USA) and was stated in percentage of the total myocardial area (34).

### ELISA method for the evaluation of cardiac enzymes

Blood after myocardial I/R injury were collected two hours later and the levels of troponin I, lactate dehydrogenase (LDH), myoglobin, and creatine kinase myocardial band (CK-MB) in serum were evaluated using a spectrophotometer via ELISA based commercial kits, which include Spinreact LDH, Coral clinical system CK-MB, and Genxbio troponin I. The kits were used according to the manufacturer's protocols. The troponin-I assay kit (Vitrosens VMD49) exhibited a lower limit of detection (LOD)/cut-off value of 0.5 ng/mL according to the manufacturer's specifications.

## Biochemical estimations

### Preparation of homogenate

Rats were sacrificed, and the hearts were cautiously excised and washed with ice-cold isotonic saline solution. The tissues were then homogenized in ice-cold phosphate buffered solution (0.1 M, pH 7.4). The resultant homogenate was centrifuged (Remi Cooling Centrifuge CPR 24) at 10,000 rpm at 4 °C for a duration of 15 min. The supernatant was collected and aliquot solutions were separated for the following biochemical assessments.

### Evaluation of pro-inflammatory parameters

In the preventive model, pro-inflammatory cytokine markers, including interleukin (IL-6), and tumour necrosis factor-alpha (TNF-α) were assessed in both myocardial tissue homogenates, and serum. In treatment model, levels of TNF-α, and IL-6 were measured in myocardial tissue only, following the usage instructions from the manufacturers. Spinreact total proteins kit was used to evaluate the total protein content. The cytokine levels in tissue samples were expressed as pg/mg protein.

### Biochemical estimations

The levels of nitrites, malondialdehyde (MDA) as well as the levels of glutathione (GSH) and superoxide dismutase (SOD) were used to evaluate oxidative stress in heart homogenates.

#### Evaluation of malondialdehyde (MDA) levels

Malondialdehyde (MDA), a chief product of lipid peroxidation, was quantitatively evaluated as thiobarbituric acid reactive substances (TBARS). In the present assay, 0.1 mL of samples was mixed individually with sodium dodecyl sulphate (0.1 mL, 8.1%), glacial acetic acid (0.75 mL 20%), thiobarbituric acid (TBA) (0.75 mL 0.8%), and distilled water (0.3 mL), and incubated at 95 °C for 1 h. After incubation, the supernatants were centrifuged at 10,000 rpm for 10 min separately. The supernatants were collected and MDA levels were evaluated at 532 nm using an Epoch-Biotek multiplate spectrophotometer (Agilent Technologies, Santa Clara, CA, USA). Tetramethoxypropane was served as the standard. Results were stated as nanomoles of MDA per milligram of protein (35).

#### Evaluation of reduced superoxide dismutase (SOD) levels

The reduced superoxide dismutase level was estimated spectrophotometrically through the epinephrine auto-oxidation at pH 10.4. For the evaluation, samples were assorted with glycine buffer (90.8 mL, 50 mM, pH 10.4) and the reaction was initiated by adding (-)-epinephrine (90.02 mL). Post 5 min of incubation, the absorbance was evaluated spectrophotometrically (Shimadzu UV-1800, Japan) at 480 nm.

#### Evaluation of the levels of reduced glutathione (GSH)

Reduced glutathione levels were estimated using the Ellman method. Aliquot equal volumes of samples and sulphosalicylic acid (5%) were assorted individually and incubated at 4 °C for 1 h. The mixtures were then centrifuged by maintaining a temperature of 4 °C at 10,000 rpm for 10 min. The clear supernatant (50 *μ*L) thus formed was mixed with phosphate buffer (450 *μ*L) and 5, 5-dithiobis-(2-nitrobenzoic acid) (1.5 mL) in phosphate buffer (0.1 M, pH 8.0). The resultant reaction mixture was then incubated at 37 °C for 10 min and the developed colour was evaluated at 412 nm using an Epoch-Biotek multiplate spectrophotometer (Agilent Technologies, Santa Clara, CA, USA). Results were given in micromole per milligram of protein.

### Statistical analysis

Statistical analyses were performed using GraphPad Prism version 5.0. Data were initially assessed for normality using the Shapiro–Wilk test and for homogeneity of variances using Levene's test. Preliminary sex-wise comparisons of the measured endpoints were performed within each experimental group to assess potential sex-related differences. No statistically significant differences were observed between male and female animals for the evaluated parameters, and no consistent sex-specific trend was identified. Therefore, data from both sexes were pooled for final statistical analysis. Since all datasets satisfied the assumptions for parametric testing (*p* > 0.05), statistical comparisons were performed using one-way ANOVA followed by Tukey's multiple comparison *post hoc* test. Outcome assessments, including infarct size quantification, ELISA measurements, and biochemical estimations, were performed by investigators blinded to treatment allocation to minimize observer bias.

## Results

### Extraction of Oxystorm from *Amaranthus* leaves

Preliminary extraction trials were carried out with different solvent systems including, ethanol, methanol, and hydroethanolic mixtures (50:50, 70:30, 80:20 v/v) to obtain a nitrate enriched extract from *Amaranthus* leaves. Following repeated trials of extraction and HPLC analyses, highest nitrate content was detected in the millipore water extract, designated as Oxystorm, which yielded 3.20% (w/w). Owing to the superior nitrate enrichment, water extract was subjected to further evaluations. Subsequently, the extract was standardised and quantified for nitrate content using a HPLC method prior to cardioprotective assessments.

### HPLC analyses of nitrates in Oxystorm and curcuminoids in turmeric extract (BCM-95)

**Figure 2:**
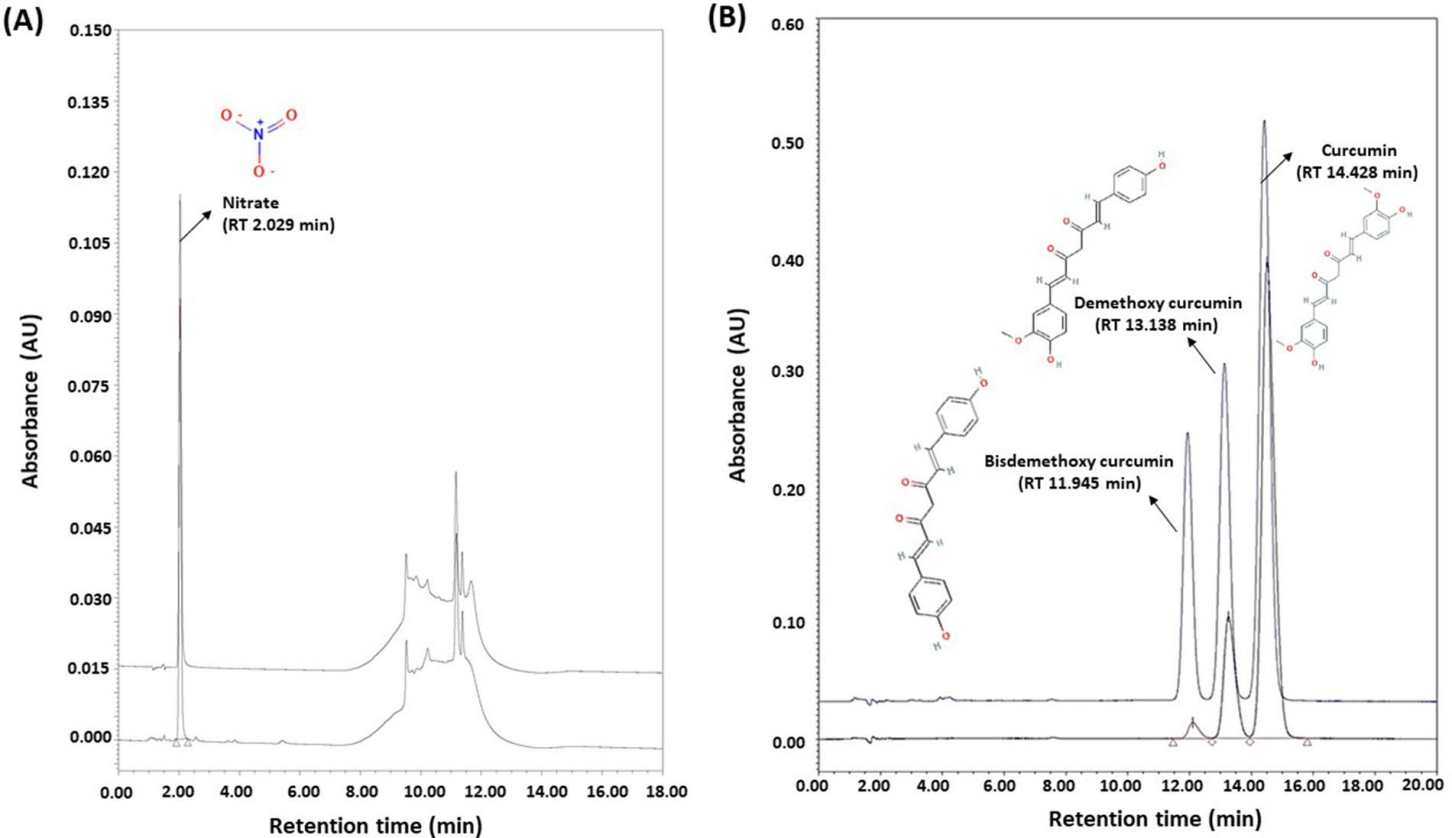
High performance liquid chromatographic analyses of Oxystorm and curcuminoid enriched turmeric extract (BCM-95). **(A)** Nitrate in Oxystorm was eluted at a retention time of 2.029 min using a gradient elution of mobile phase A [tetrabutyl ammonium hydroxide (TBAH) buffer, pH 2.5], B (acetonitrile), and C (methanol). **(B)** The curcuminoids, curcumin, demethoxy curcumin, and bisdemethoxy curcumin was eluted at 14.428 min, 13.138, and 11.945 min using an isocratic elution of acetonitrile and 0.1% formic acid in water (45:55, v/v).

Simple and efficient high-performance liquid chromatography methods were employed for the evalution of nitrate content in Oxystorm and curcuminoids in turmeric extract (BCM-95). For the analysis of nitrate, a gradient elution of different ratios of mobile phase A (TBAH buffer, pH 2.5), B (acetonitrile), and C (methanol) at a flow rate of 0.1 mL/min resulted in a sharp and well resolved peak at a retention time of 2.029 min against reference standard (Figure 2A). However, an isocratic solvent system consisting of acetonitrile and 0.1% formic acid in water (45:55, v/v) with a flow rate of 1.0 mL/min resulted in the elution of curcumin, demethoxy curcumin, and bisdemethoxy curcumin at 14.428, 13.138, and 11.945 min respectively (Figure 2B). Quantification of nitrate in Oxystorm and curcuminoids in turmeric extract was achieved using the respective analytical conditions. Subsequently, the contents were calculated using Equations (1) and (4), respectively. The nitrate content in Oxystorm was determined as 9.06% (w/w) whereas the curcuminoid content in turmeric extract was found to be 89.10% (w/w).

### Total phenolic and flavonoid constituents of turmeric extract (BCM-95)

**Figure 3:**
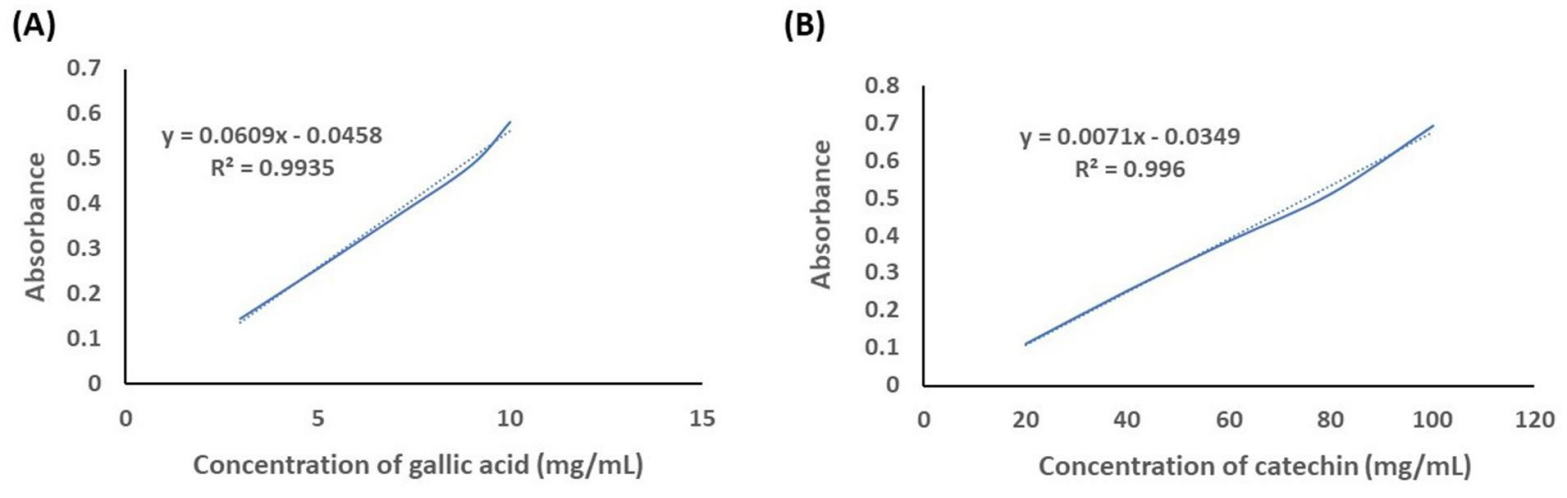
Calibration graphs plotted for evaluating total phenolic and flavonoid contents in curcuminoid enriched turmeric extract (BCM-95). **(A)** Calibration plot of gallic acid, constructed by plotting concentration of gallic acid (mg/mL) against corresponding absorbance. The obtained regression equation was *y* = 0.0609*x* − 0.0458, with a correlation coefficient *R*^2^ = 0.9935. **(B)** Represents the plot of calibration in which concentration of catechin was plotted against absorbance. The corresponding resultant regression equation was *y* = 0.0071*x* − 0.0349, with a correlation coefficient value of 0.9960.

Total phenolic and total flavonoid contents are widely used to assess the antioxidant potential as well as the phytochemical diversity of plant extracts. In the present study, the contents were calculated using the calibration graphs plotted separately with concentrations of gallic acid (Figure 3A) and catechin (Figure 3B) reference standards. The total phenolic and flavonoid contents were evaluated to be 90 g gallic acid equivalents/100 g extract and 3 g catechin equivalents/100 g extract, respectively. It is observed that high phenolic and flavonoid contents in turmeric extract may therefore be attributed primarily to the abundance of curcuminoids and other polyphenolic constituents such as flavonoids present in rhizomes. Polyphenolic compounds possess promising antioxidant properties due to their ability to scavenge reactive oxygen species and to reduce oxidative stress (36). Moreover, the presence of these phytochemicals supports the use of turmeric in the alleviation of inflammatory diseases, and oxidative stress conditions (37, 38).

### Protective effect on cardiac myocytes

**Figure 4:**
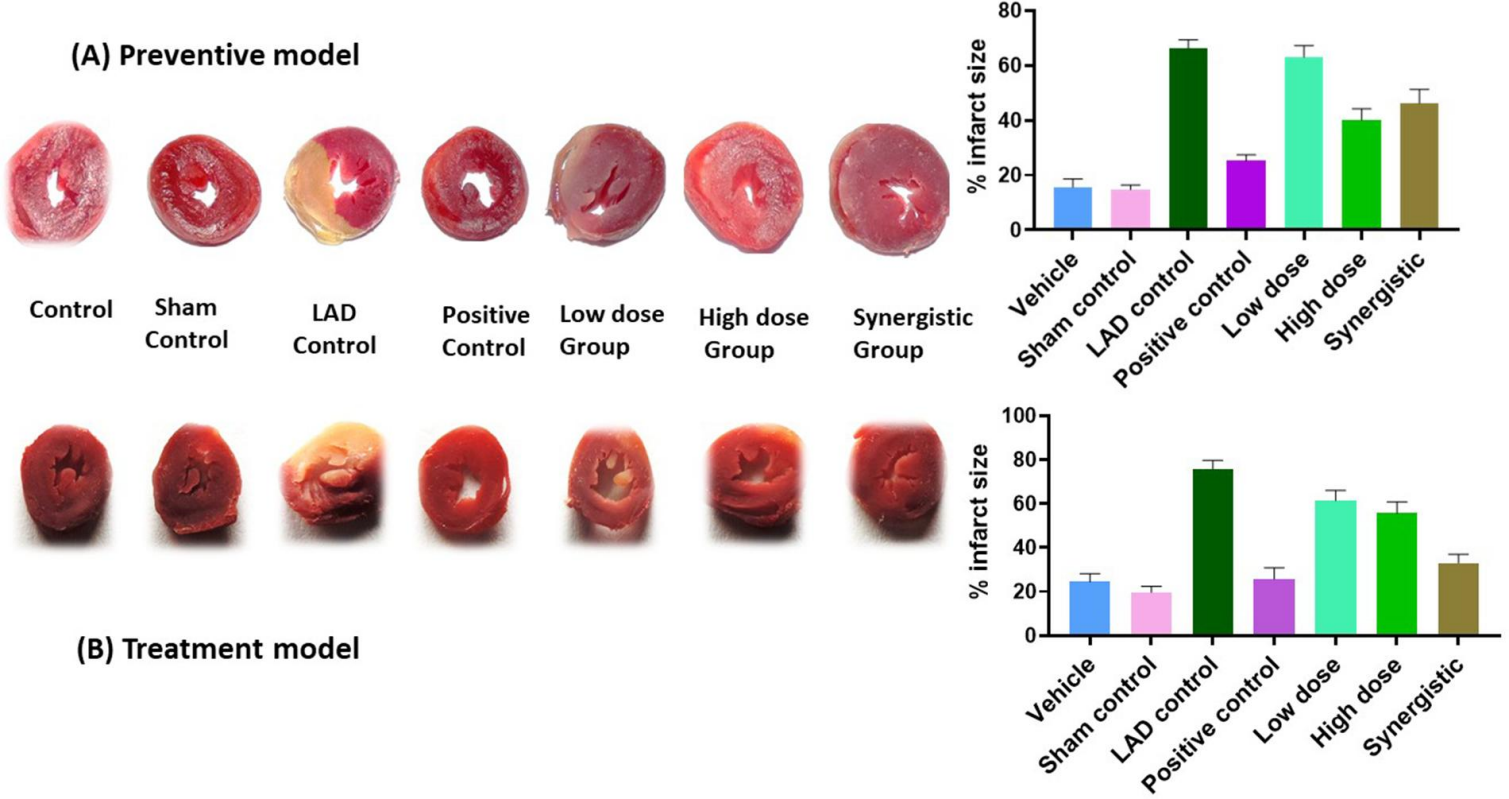
Effect of Oxystorm and its synergistic combination with curcuminoids enriched turmeric extract (BCM-95) on infarct size of heart homogenates on 31st day (*n* = 56). **(A)** and **(B)** Represents the impact of test formulations in pretreatment and therapeutic models. **(**Significant difference was observed for positive control after 4 weeks of intervention compared to LAD control. In contrast, a significant increase in % infarct size (***p < 0.001) for LAD control was observed when compared to Sham. All values were expressed in mean ± SEM.

The cardioprotective effect of test formulations was evaluated on myocardial I/R injury model, in which 2,3,5-triphenyltetrazolium chloride (TTC) staining was used to assess myocardial infarct area and gross tissue viability. As depicted in Figures 4A,B, the left anterior descending coronary artery ligation resulted in prominent increase in myocardial infarction area, which reached 66.38 ± 2.97% and 75.90 ± 3.83% in pre-treatment and post-treatment models respectively in comparison with the control group. The observations confirm the successful induction of myocardial damage through the ligation of LAD coronary artery method. However, pre-treatment with the test formulations considerably reduced the infarct size, with the most pronounced effects observed in high dose Oxystorm group and synergistic group (***p < 0.001). Similarly, substantial decrease was observed in post treatment with test formulations, particularly in synergistic group (***p < 0.001) compared with high dose Oxystorm group (**p < 0.01). The positive control also showed a noteworthy decrease in infarct percentage (***p < 0.001) in both models. Here our results elucidated the synergic cardioprotective effect of our test formulation with curcuminoids enriched turmeric extract against myocardial ischemia *in vivo*.

**Figure 5:**
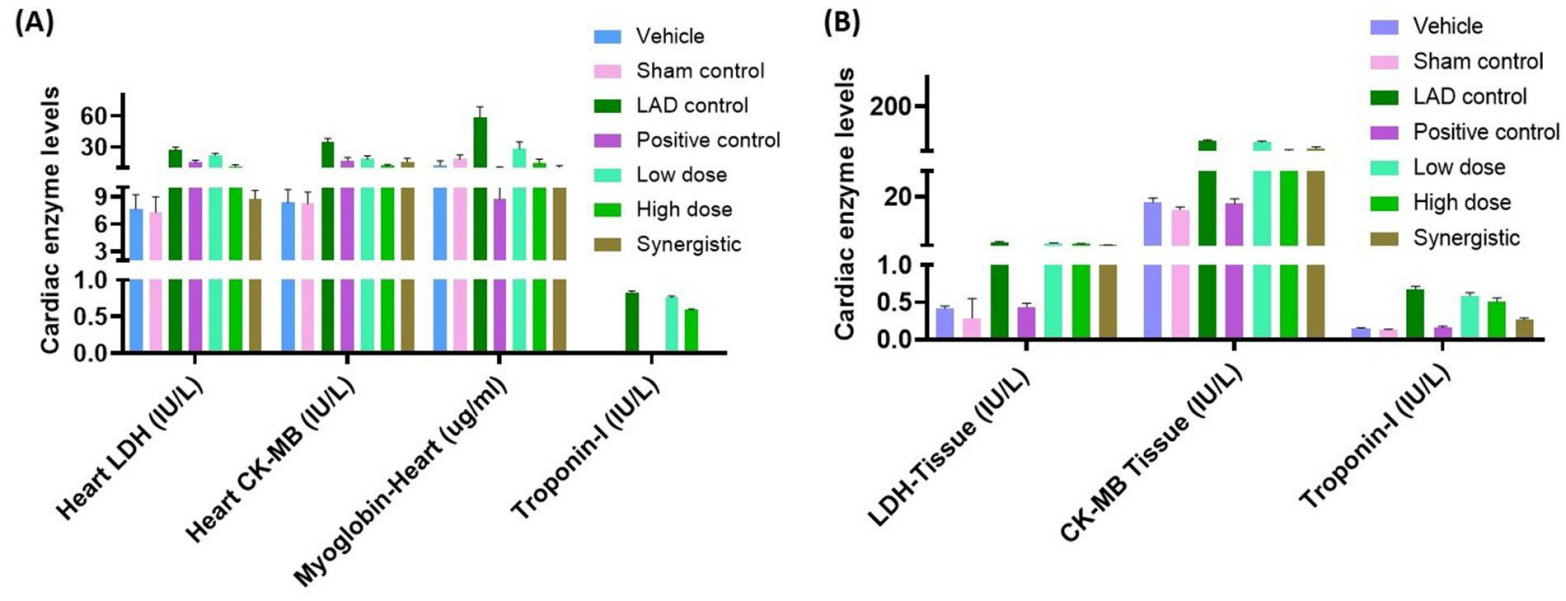
Effect of Oxystorm and its synergistic combination with curcuminoids enriched turmeric extract (BCM-95) on LDH level of heart homogenates on 31st day (*n* = 56). **(A)** Represents elevated levels of LDH, CK-MB, and myoglobin in ligated rats (26.90 ± 3.15 IU/L for LDH, 34.53 ± 3.80 IU/L for CK-MB, and 57.88 ± 10.51 *μ*g/mL for myoglobin) compared with the control group. **(B)** Depicts the effect of test formulations on inflammatory biomarkers. Levels of LDH and CK-MB in cardiac tissue were remarkably increased in rats of I/R group. Test formulations reduced the levels of LDH and CK-MB across all doses, but substantial attenuation was observed in synergistic treatment. Significant difference was observed for positive control after 4 weeks of intervention compared to LAD control. All values were expressed in mean ± SEM.

The important biomarkers of myocardial damage including LDH, CK-MB, and myoglobin in heart and troponin-I were monitored to evaluate the range of cardiac injury. The enzymes (CK-MB and LDH) were released from myocardial cells after ischemic injury. As given in Figure 5A, LDH, CK-MB, and myoglobin levels were significantly elevated in ligated ones (26.90 ± 3.15 IU/L for LDH, 34.53 ± 3.80 IU/L for CK-MB, and 57.88 ± 10.51 *μ*g/mL for myoglobin) related to the control group. Oxystorm preconditioning reduced all assessed biomarker levels at all tested doses, with a significant attenuation at the high dose of 90 mg/kg (*** *p* < 0.001) and in synergistic treatment group (***p < 0.001) relative to the I/R group. Troponin-I levels were detected in both low- and high-dose Oxystorm groups; however, in the synergistic pre-treatment group, the levels were below the assay's lower limit of detection (0.5 ng/mL) compared with the LAD control group (Figure 5A). Therefore, this finding should be interpreted as a marked reduction in circulating troponin-I rather than complete absence of myocardial injury. The observations indicate potential cardioprotective effects of the synergistic formulation containing curcuminoids-enriched turmeric extract and suggest attenuation of myocardial injury.

In the treatment model (Figure 5B), the levels of CK-MB and LDH in cardiac tissue were markedly elevated in I/R group (2.23 ± 0.22 IU/L for LDH, and 84.3 ± 4.66 IU/L for CK-MB) related to the control groups. Treatment with test formulations reduced the levels of LDH and CK-MB across all doses, however, substantial reduction was detected in synergistic treatment group relative to the I/R group. The synergistic combination reduced LDH, and CK-MB levels in heart tissue, the typical biomarkers of myocardial dysfunction and cardiac arrest. Additionally, a significant elevation in LDH (***p < 0.001) was observed in LAD control compared to sham. Troponin I levels were also reduced significantly in all treatment groups compared to the control group, further supports the cardioprotective effects of the studied formulations.

### Effect on pro-inflammatory markers

**Figure 6:**
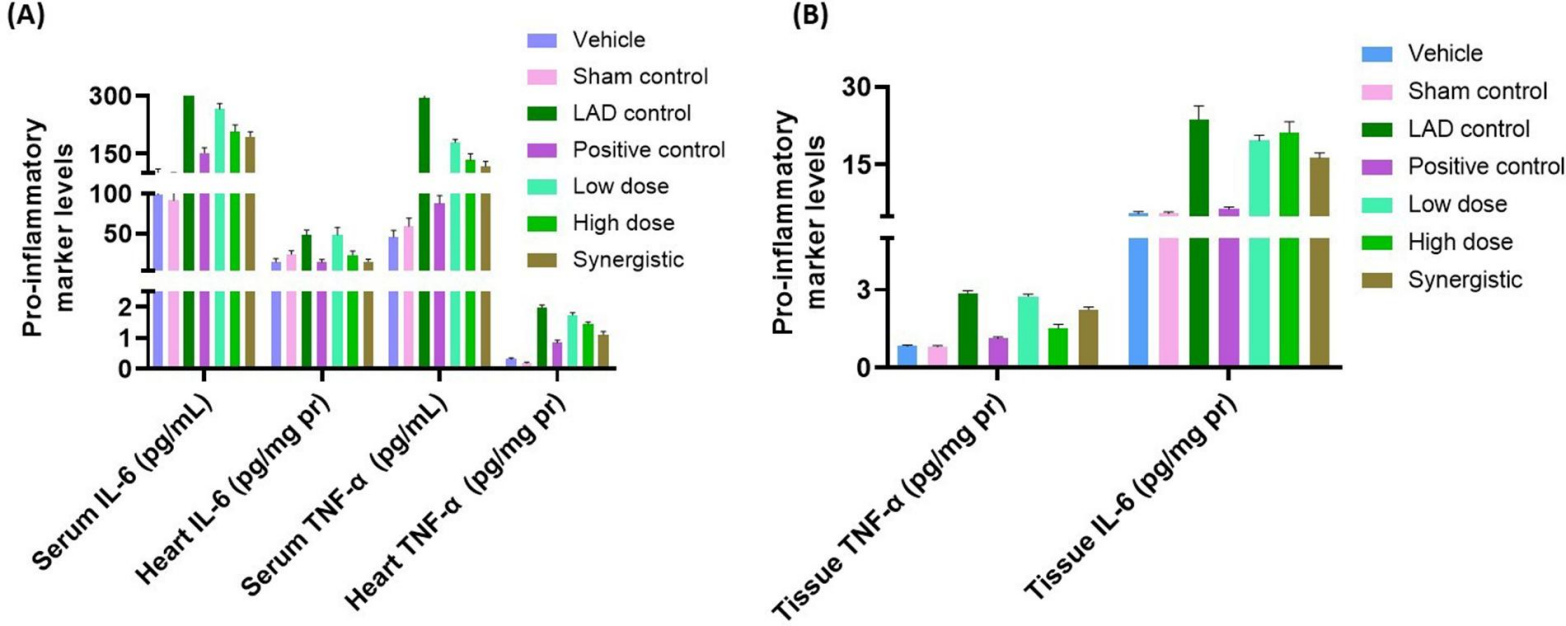
Effect of Oxystorm and its synergistic combination with curcuminoids enriched turmeric extract (BCM-95) on IL-6 and TNF- *α* levels on 31st day (*n* = 56). **(A),(B)** Portrays the change of IL-6 and TNF-α concentration of all tested rats in both serum and cardiac tissue in both models respectively.

Cytokines, an assorted group of signaling proteins, play a central role in mediating the inflammatory response during the progression of ischemia/reperfusion injury. The levels of inflammatory cytokines following MI/R were quantified using ELISA. As shown in Figures 6A,B, the concentrations of IL-6 and TNF-α in both serum and cardiac tissue were remarkably increased in all experimental groups following I/R injury compared to the control group, which indicated a pronounced inflammatory response. Pre-treatment with test formulations significantly reduced the cytokines release, demonstrating its anti-inflammatory effects. As expected, cytokine suppression occurred dose dependently, with the combination synergistic group producing reductions comparable to those observed in the positive control groups (***p < 0.001). Moreover, administration with test formulation in the synergistic group notably attenuated the cytokines release in tissue samples when related with LAD control. Interestingly, a commendable decrease of TNF-α was observed in high dose group compared to I/R group (***p < 0.001), which indicated a dose-dependent anti-inflammatory effect.

### Effect on oxido-nitrosative stress

**Figure 7:**
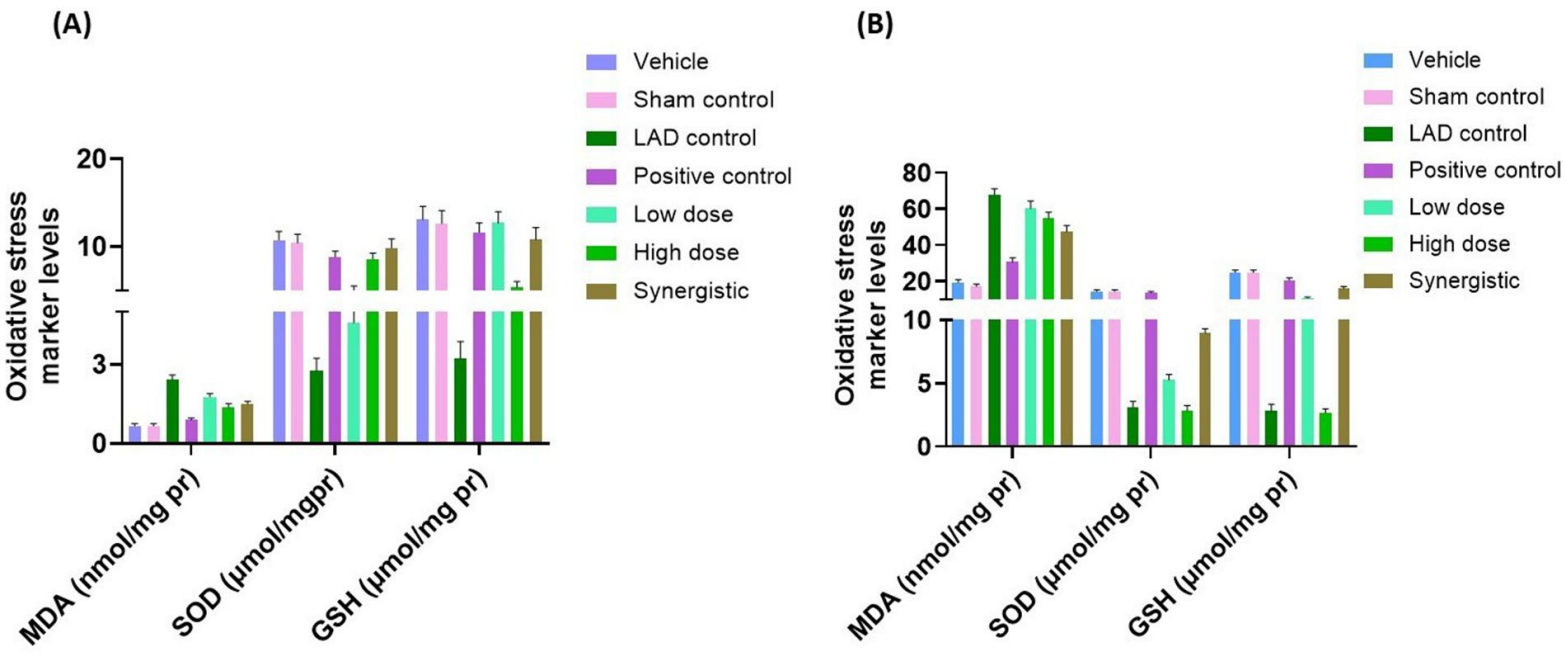
Effect of Oxystorm and its combination with curcuminoids enriched turmeric extract (BCM-95) on MDA, SOD and GSH levels on 31st day (*n* = 56). **(A),(B)** depicts the change of MDA, SOD, and GSH levels on all tested rats in both models respectively.

MDA, GSH, and SOD serve as key markers of oxidative stress. As shown in Figures 7A,B, corresponding to the preventive and treatment models respectively, the results showed that the level of MDA decreased dose dependently at both dosages (**p < 0.01 for low and ***p < 0.001 for high) following treatment with the test formulation. The synergistic group also displayed a noteworthy reduction (***p < 0.001) compared to I/R rats. The activities of SOD expressively amplified in high dose group (***p < 0.001) and synergistic group (***p < 0.001) relative to I/R rats. Likewise, GSH levels were markedly elevated in group with lower dosage (***p < 0.001) and remarkably elevated in synergistic group (***p < 0.001) compared with I/R group. These findings noted that LAD induced I/R damage markedly elevated oxidative stress, as evidenced by elevated MDA levels and impaired endogenous antioxidant defense. Moreover, treatment with the test formulation particularly at high and synergistic doses, significantly attenuated oxidative damage by reducing lipid peroxidation (MDA), and restoring endogenous antioxidant defense systems (SOD and GSH).

## Discussion

The current study established the role of a nitrate enriched dietary herbal formulation, Oxystorm and its combination with curcuminoids enriched turmeric extract (BCM-95) effectively mitigated the damage caused of MI and its reperfusion via left anterior descending coronary artery (LAD) ligation. We identified a prominent elevation in myocardial infarction size, LDH, CK-MB, myoglobin, and inflammatory biomarkers, and induced oxidative stress on cardiac cells after the left anterior descending coronary artery ligation. Infarct size, a key indicator in assessing the severity of MI/R injury was significantly reduced following the administration of a series of test formulations, in which Oxystorm demonstrated a dose-dependent effect (Figure 4).

Moreover, the reduction in CK-MB level was measured to determine whether test formulation effectively mitigated myocardial injury. Similarly, LDH, a cytoplasmic enzyme released during cardiac injury, was significantly reduced succeeding treatment, reflect the reduced cellular damage. Overall, the findings further confirmed the cardioprotective effect of synergistic combination and Oxystorm on cardiac myocytes in *in vivo* preventive model, which decreased the biomarker enzymes associated with myocardial cell injury (Figure 5). The promising anti-inflammatory potential by the suppression of TNF-α and IL-6 cytokine levels in a dose dependent manner (Figure 6), may be attributed by the antioxidant constituents present in the formulations, which probably inhibited reactive oxygen species mediated activation of inflammatory pathways during reperfusion. These findings suggest that the test formulations possess marked cardioprotective potential against ischemic injury by modulating the inflammatory pathway.

Malondialdehyde is a well-established lipid peroxidation marker resulting from the uncontrolled production of reactive oxygen species (ROS). In contrast, superoxide dismutase is a prominent endogenous antioxidant enzyme, which scavenge ROS and protect cell from oxidative damage. GSH, a glutamate tripeptide, serves as an essential antioxidant and also function as a neuromodulator in the central nervous system, which serves as the most powerful endogenous antioxidant in the cardiovascular system, contributing significantly in detoxifying xenobiotics and scavenging reactive oxygen species. Previous reports have suggested that disruption of glutathione system plays a critical role in the pathogenesis of myocardial injury (39). An imbalance between oxidative and anti-oxidative mechanisms leads to the oxidative damage. In the present study, the test formulations dose dependently reduced MDA levels, especially at 90 mg/kg Oxystorm group in the preventive model and synergistic group in the treatment model (Figure 7). Taken together with these experimental findings, the results suggest that the test formulation and its synergistic combination were associated with attenuation of oxidative stress and reduced indicators of myocardial injury during ischemia-reperfusion injury *in vivo.*

**Figure 8:**
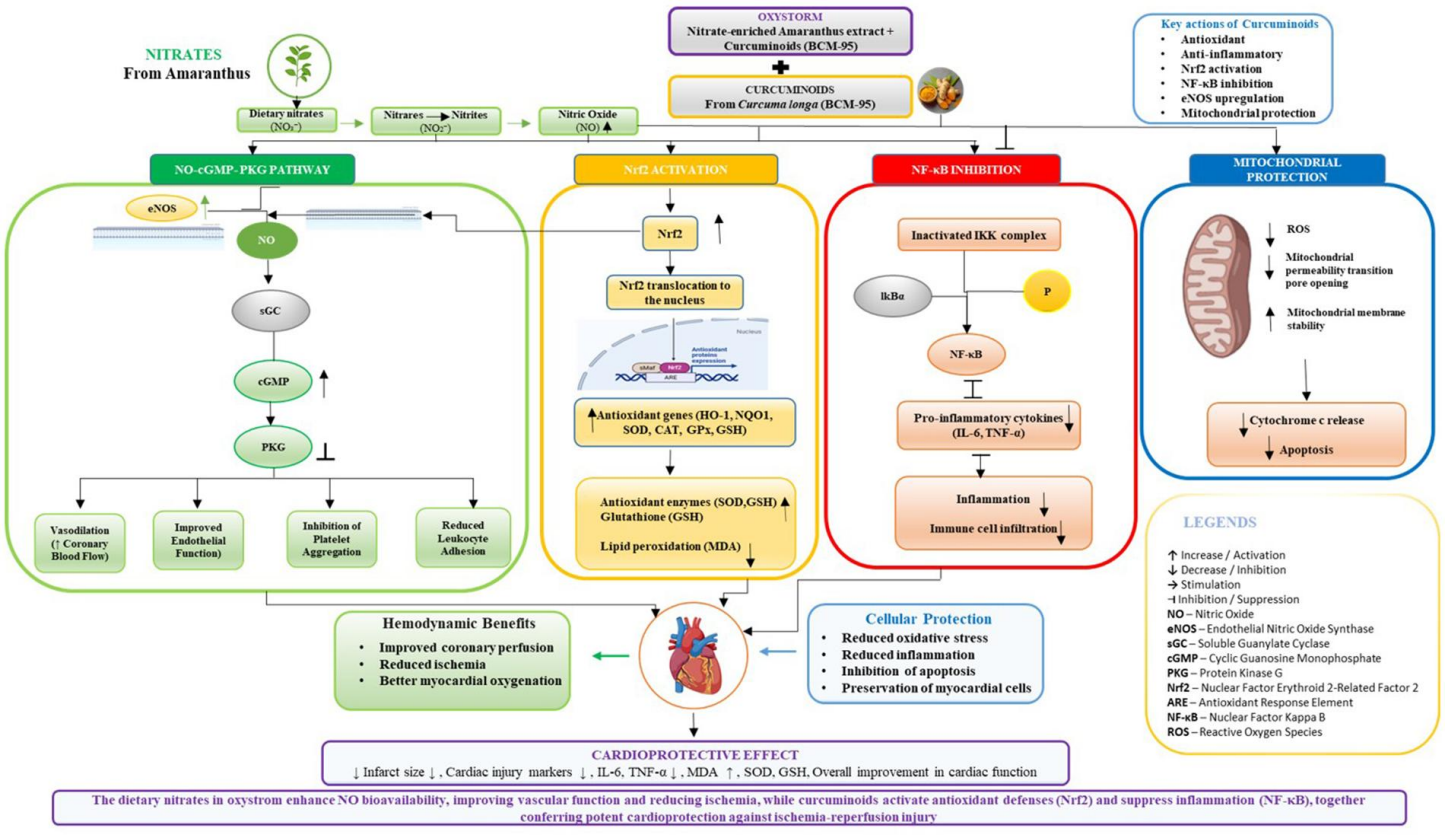
Proposed cardioprotective mechanisms of Oxystorm and curcuminoid-enriched turmeric extract (BCM-95) against myocardial ischemia–reperfusion (MI/R) injury. Dietary nitrates present in Oxystorm may contribute to nitrate-derived nitric oxide (NO)-related signaling pathways, potentially involving the NO–cGMP–PKG axis, which has been associated with vasodilation, improved endothelial function, and enhanced coronary perfusion. Curcuminoids may activate Nrf2-mediated antioxidant defenses, leading to increased expression of antioxidant enzymes and reduced oxidative stress, while also suppressing NF-*κ*B signaling and the production of pro-inflammatory cytokines (TNF-α and IL-6). In addition, mitochondrial protection may help limit apoptosis and preserve cardiomyocyte viability. Collectively, these complementary mechanisms may contribute to reduced infarct size, attenuation of oxidative stress and inflammation, and improved cardiac function following MI/R injury. The figure was created by the authors using Microsoft PowerPoint. Graphical elements were adapted from publicly available online resources and assembled for illustrative purposes.

Imbalances in vascular smooth muscle character and alterations in the structural components of the arterial wall are key contributors of increased arterial stiffness (40), driven by reduced nitric oxide bioavailability along with increased oxidative stress and inflammation (41). Beyond marked levels of nitrate content and its contributing role in cardiac health, *Amaranthus* has been reported as a potential dietary adjunct for improving cardiovascular risk profiles by alleviating total cholesterol, low density lipoprotein, triglyceride levels, as well as inhibiting platelet activation (42, 43). Curcumin, a signature compound among curcuminoids and a signature phenolic compound found in turmeric, has been reported to modulate nitric oxide-related vascular responses and reduce oxidative stress in experimental models (44). Moreover, in a preclinical study (45), it is demonstrated that supplementation with curcumin for a period of four weeks enhanced conduit artery endothelial function in aged male mice to those of younger ones. The change was associated with improved nitric oxide-related vascular responses and reduced vascular oxidative stress. Further supplementation reduced aortic stiffening, as evidenced by aortic pulse wave velocity values approaching those observed in young adult mice. These findings highlight the age-related therapeutic potential of curcumin in mitigating arterial dysfunction. Furthermore, the inverse relation between curcumin and aortic stiffness has been linked to its potential bioavailability, which plays crucial role in the reduction of cardiovascular mortality (46). These mechanisms could potentially contribute to the enhanced cardioprotective effects observed in the high-dose Oxystorm and synergistic treatment groups in the present study. However, nitric oxide bioavailability was not directly measured in the current investigation, and the involvement of NO-mediated signaling is inferred from the nitrate-enriched composition of Oxystorm and supporting literature. In the present study, both male and female rats were included with equal distribution across experimental groups. In the present study, both male and female rats were included with equal distribution across experimental groups. Preliminary sex-wise comparisons were performed and did not reveal significant differences in the measured endpoints; therefore, data from both sexes were pooled for analysis. Since estrogen and other sex hormones are known to influence vascular function, oxidative stress responses, inflammatory signaling, and myocardial ischemia-reperfusion injury, their potential contribution to the observed cardioprotective effects cannot be excluded. Studies have demonstrated that estrogen may exert endogenous cardioprotective effects by modulating nitric oxide signaling, mitochondrial function, and antioxidant defense systems. Therefore, future investigations incorporating sex-stratified analyses and hormonal profiling are warranted to better understand possible gender-dependent variations in response to Oxystorm and its synergistic combination with curcuminoids-enriched turmeric extract. Further evidence suggests that the synergistic cardioprotective activity of nitrate-enriched formulations and curcumin-based interventions may involve pathways associated with nitric oxide signaling and the Nrf2/ARE antioxidant defense system. Nrf2 is recognized as a major transcriptional regulator of cellular antioxidant responses and plays a critical role in protection against oxidative stress-mediated myocardial injury. Curcumin has been widely reported to activate Nrf2-associated antioxidant signaling, while nitric oxide-related cytoprotective responses may interact with redox-sensitive pathways involved in vascular and myocardial protection (4). Therefore, the enhanced cardioprotective efficacy observed in the Oxystorm and BCM-95 combination group may be partly associated with nitrate/NO-related pathways, antioxidant defense mechanisms, and inflammatory signaling pathways. Nevertheless, direct molecular evaluation of Nrf2/ARE-associated signaling was not performed in the present study and requires further mechanistic investigation. Overall, the findings indicate potential cardioprotective effects of the synergistic combination of Oxystorm in the *in vivo* preventive model, as evidenced by reductions in biomarkers associated with myocardial injury. The markedly reduced troponin-I levels observed in the synergistic group may indicate substantial attenuation of myocardial injury. Troponin-I levels in the synergistic pre-treatment group were below the lower limit of detection of the assay; therefore, this finding should be interpreted as a marked reduction in circulating troponin-I rather than complete absence of myocardial injury. However, low basal biomarker release below the assay's detection limit cannot be excluded. The overall cardiovascular efficacy of the herbal combination might be attributed by the synergistic interaction between nitrate and polyphenol constituents. These findings were supported by both HPLC as well as spectrophotometric methods, which confirmed the existence of significant levels of signature phyto compounds in the formulation. Although the present study did not directly evaluate molecular signaling mediators, the observed reductions in infarct size, inflammatory cytokines, and oxidative stress markers suggest the involvement of multiple cardioprotective pathways. Based on the known biological activities of dietary nitrates and curcuminoids reported in the literature, a proposed mechanistic framework underlying the cardioprotective effects of Oxystorm and its synergistic combination with turmeric extract was illustrated in Figure 8.

As shown in Figure 8, a hypothetical mechanistic framework based on literature evidence is proposed. Nitrate-derived nitric oxide may enhance vascular function through NO–cGMP–PKG signaling, while curcuminoids may exert antioxidant and anti-inflammatory effects via Nrf2 activation and inhibition of NF-*κ*B-mediated cytokine production. These pathways were not directly evaluated in the present study. These complementary mechanisms may collectively contribute to the favorable biochemical and infarct-size outcomes observed following myocardial ischemia–reperfusion injury in the present study. Similarly, although Oxystorm is a nitrate-enriched formulation and the observed cardioprotective effects are consistent with a potential role of nitrate-derived nitric oxide signaling, nitric oxide bioavailability, nitrate/nitrite metabolites, eNOS activity, and downstream signaling mediators were not directly quantified. Therefore, the proposed involvement of nitric oxide remains inferential and should be confirmed in future mechanistic studies.

## Future research

Targeted phytochemical characterisation of Oxystorm and BCM-95 was performed by quantifying nitrate and curcuminoid content, along with total phenolic and flavonoid contents. However, comprehensive metabolite profiling, including broader HPLC or LC-MS-based chemical fingerprinting, was beyond the scope of the present study and will be addressed in future work. Although the present study demonstrated significant cardioprotective effects of Oxystorm and its synergistic combination with curcuminoid enriched turmeric extract, as assessed by biochemical, inflammatory, oxidative stress, and infarct size markers, several advanced investigations remain warranted for a deeper mechanistic understanding. Future studies should incorporate echocardiographic hemodynamic monitoring and cardiac functional assessments, including ejection fraction, fractional shortening, ventricular remodeling, and myocardial contractility, to better characterize functional recovery following ischemia-reperfusion injury. In addition, detailed histopathological evaluations, including Hematoxylin & Eosin (H&E) and Masson's Trichrome staining, would further validate myocardial structural preservation and fibrosis attenuation at the cellular level.

Furthermore, molecular investigations targeting oxidative stress and inflammatory signaling pathways would strengthen the mechanistic interpretation of the observed cardioprotective effects. Evaluation of key proteins and transcription factors, such as nuclear factor erythroid 2-related factor 2 (Nrf2), nuclear factor-kappa B (NF-*κ*B), endothelial nitric oxide synthase (eNOS), apoptotic markers, and mitochondrial stress-associated pathways, using Western blotting or immunohistochemical analyses, may provide comprehensive insight into the underlying molecular mechanisms. Although TTC staining and biochemical analyses provided substantial evidence of cardioprotection, detailed histopathological and fibrosis-associated evaluations were beyond the scope of the present investigation and warrant further study.

## Conclusions

The present study highlights the potential dietary interventions in managing cardiovascular health, which demonstrated noteworthy protection in contradiction of myocardial ischemia-reperfusion damage in Wistar rats may be contributed by the phytoconstituents present in test formulations as evidenced from HPLC and spectrophotometric analyses. Treatments significantly reduced LDH, CK-MB, and troponin I levels, decreased infarct size, suppressed pro-inflammatory cytokine levels, and alleviated oxidative stress while conserving antioxidant defenses. Overall, preconditioning with the high-dose dietary nitrate-enriched *Amaranthus* extract, Oxystorm, and its combination with curcuminoids enriched turmeric extract was associated with improved biochemical and infarct-size indicators of cardioprotection following I/R injury-induced cardiac damage through the inhibition of inflammatory biomarkers and oxidative stress markers, confirming their prominent cardioprotective potential compared to low dose Oxystorm group, which showed only moderate activity. These beneficial effects may partly be associated with nitrate/NO-related pathways linked to the nitrate-rich composition of Oxystorm, either alone or in combination with curcuminoids; however, these mechanisms were not directly verified in the present study. However, direct evaluation of nitric oxide bioavailability and related signaling mediators was beyond the scope of the present study.

Although troponin-I levels in the synergistic pre-treatment group were below the assay detection limit, this finding indicates a marked reduction in myocardial injury biomarkers rather than complete prevention of myocardial damage. Although TTC staining effectively demonstrated a reduction in infarct size and myocardial injury, detailed histopathological analyses, such as Hematoxylin & Eosin (H&E) and Masson's Trichrome staining, were not performed in the present study. Therefore, cellular-level alterations, including myocardial fiber disarray, edema, inflammatory infiltration, and collagen deposition/fibrosis, could not be evaluated. Accordingly, while TTC staining and biochemical assessments demonstrated reduced infarct size and favorable cardioprotective biomarker profiles, direct histological evidence of myocardial tissue preservation was not established in the present study. Future studies involving comprehensive histomorphological assessment are warranted to further validate the cardioprotective effects of the tested formulations. Additionally, although both sexes were included in the study, sex hormone-related influences, particularly estrogen-mediated cardioprotective effects, were not separately evaluated and may represent a potential confounding factor. Conclusively, both Oxystorm and its BCM-95 combination may manage cardiac ischemic reperfusion injury in the forthcoming therapeutics. Further mechanistic investigations, including direct quantification of nitric oxide metabolites, assessment of nitrate/nitrite balance, and evaluation of eNOS-related signaling pathways, are warranted to determine whether nitric oxide contributes to the observed cardioprotective effects.

## Data Availability

The original contributions presented in the study are included in the article/Supplementary Material, further inquiries can be directed to the corresponding author/s.
