# Supplementary figures and images for: Assessment of cardioprotective activity of a nitrate enriched food-based polyherbal formulation using experimental myocardial infarction in rats
Source: Front Cardiovasc Med. 2026 Jul 10;13:1857264. doi: 10.3389/fcvm.2026.1857264 (PMC13395918; doi:10.3389/fcvm.2026.1857264)

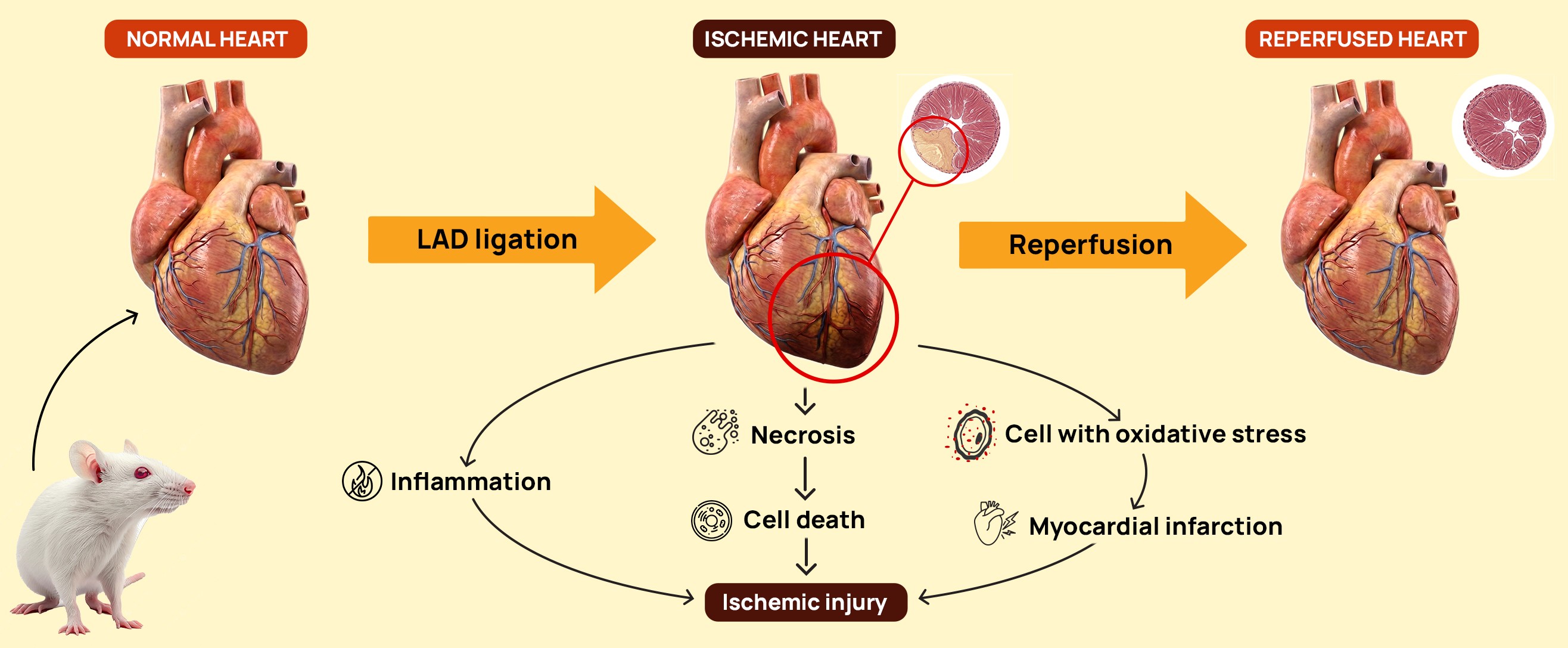

Supplement: Supplementary file 1 [file Image1.jpeg]
